# Cug2 is essential for normal mitotic control and CNS development in zebrafish

**DOI:** 10.1186/1471-213X-11-49

**Published:** 2011-08-15

**Authors:** Hyun-Taek Kim, Ju-Hoon So, Seung-Hyun Jung, Dae-Gwon Ahn, Wansoo Koh, Nam-Soon Kim, Soo-Hyun Kim, Soojin Lee, Cheol-Hee Kim

**Affiliations:** 1 Department of Biology, Chungnam National University, Daejeon, South Korea; 2 Department of Microbiology, Chungnam National University, Daejeon, South Korea; 3 Genome Research Center, Korea Research Institute of Bioscience and Biotechnology, Daejeon, 305-764, South-Korea; 4 Biomedical Sciences, St. George's Medical School, University of London, London, SW17 0RE, UK

## Abstract

**Background:**

We recently identified a novel oncogene, Cancer-upregulated gene 2 (CUG2), which is essential for kinetochore formation and promotes tumorigenesis in mammalian cells. However, the in vivo function of CUG2 has not been studied in animal models.

**Results:**

To study the function of CUG2 in vivo, we isolated a zebrafish homologue that is expressed specifically in the proliferating cells of the central nervous system (CNS). Morpholino-mediated knockdown of *cug2 *resulted in apoptosis throughout the CNS and the development of neurodegenerative phenotypes. In addition, *cug2*-deficient embryos contained mitotically arrested cells displaying abnormal spindle formation and chromosome misalignment in the neural plate.

**Conclusions:**

Therefore, our findings suggest that Cug2 is required for normal mitosis during early neurogenesis and has functions in neuronal cell maintenance, thus demonstrating that the cug2 deficient embryos may provide a model system for human neurodegenerative disorders.

## Background

Cancer-upregulated gene 2 (CUG2) is known to be differentially expressed in multiple human cancer tissues including the ovary, liver, lung, intestines and pancreas [1]. Mammalian cells overexpressing CUG2 showed hallmarks of neoplasmic transformation *in vitro*, such as increased cell proliferation, migration, invasion, anchorage-independent growth and tumor formation in nude mice, similar to the effects of the H-ras oncogene [1].

Recently, CUG2 was shown to interact with CENP-T and CENP-A, essential components of the nucleosome complex located at the centromere, and was hence named centromere protein W (CENP-W) [2,3]. The centromere is involved in sister chromatid cohesion and the attachment of spindle microtubules, and is thus responsible for accurate chromosome segregation during mitotic and meiotic cell division [4]. CENP-A, a histone H3-like core protein, is required for the recruitment of many constitutive centromere components as well as transient kinetochore components [5,6]. We and others have reported that CUG2/CENP-W forms a DNA-binding complex together with the CENP-T and CENP-A as part of the centromere chromatin structure [2,3]. SiRNA-mediated knockdown of CUG2/CENP-W in HeLa cells caused defective mitosis characterized by multipolar spindle formation as well as chromosomal misalignment and hypercondensation, resulting in mitotic arrest [2,3]. However, the *in vivo *function of CUG2 has not been studied in animal models.

To elucidate the endogenous function of CUG2 *in vivo*, we investigated the expression patterns and potential roles of *cug2 *in zebrafish during early embryogenesis. Our results indicate that Cug2 is essential for normal mitosis and CNS development, and that loss of Cug2 function lead to neurodegenerative phenotypes.

## Results

### Identification of the zebrafish *cug2 *homologue

**Figure 1:**
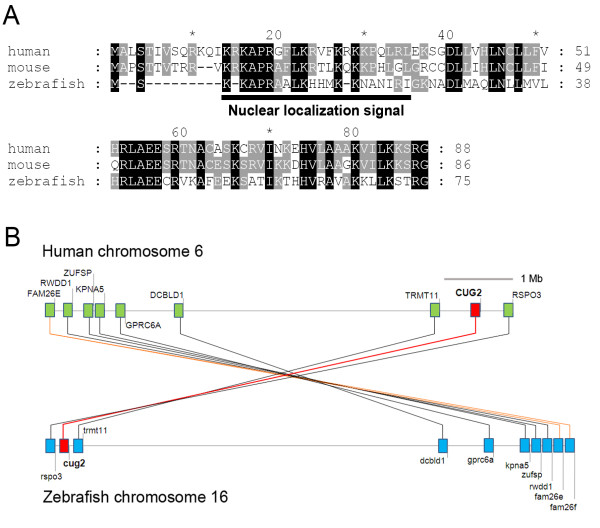
**Amino acid comparison and synteny analysis of *CUG2 *gene**. **A**. Clustal X alignment of CUG2 amino acid sequences of vertebrate homologues, with identical residues marked in black. **B**. Synteny between *cug2 *on zebrafish chromosome 16 and *CUG2 *on human chromosome 6. Using Synteny Database, synteny in the vicinity of *cug2 *gene were analyzed using zebrafish (Zv8) as the source genome and human (GRCH37) as the outgroup. The approximate position of *cug2 *is marked in red. Orthologue pairs of genes are connected by lines. Non-paired genes are not shown. The synteny occurs between the segments of zebrafish chromosome 16 and human chromosome 6 that contain the *CUG2 *gene.

A zebrafish *cug2 *homologue was isolated from a 24 hpf embryonic cDNA library. The zebrafish *cug2 *(Genbank: XM_683789) is composed of 3 exons encoding 75 amino acids. The nuclear localization signal in the *N*-terminus is highly conserved among CUG2 homologues. Clustal × analysis indicates that the zebrafish Cug2 amino acid sequence shows 58% and 62% similarity to human (Genbank: AY902475) and mouse (Genbank: XP_488549), respectively (Figure 1A). To more precisely confirm the evolutionary conservation of *CUG2*, we investigated the distribution of genes located adjacent to the *CUG2 *locus on chromosomes in zebrafish and human using the online program Synteny Database [7]. Two genes, *TRMT11 *and *RSPO3*, were located close to each other on human chromosome 6 near *CUG2*, and their zebrafish orthologues were located in the vicinity of *cug2 *on chromosome 16 (Figure 1B), indicating that zebrafish *cug2 *was an orthologue of human *CUG2*. The predicted fish *cug2 *sequence in the NCBI database (Genbank: XM_704089) suggests an isoform with alternative splicing of exon 3, and no additional isoforms were detected by RT-PCR. In addition, no evidence of a second orthologue was found.

**Figure 2:**
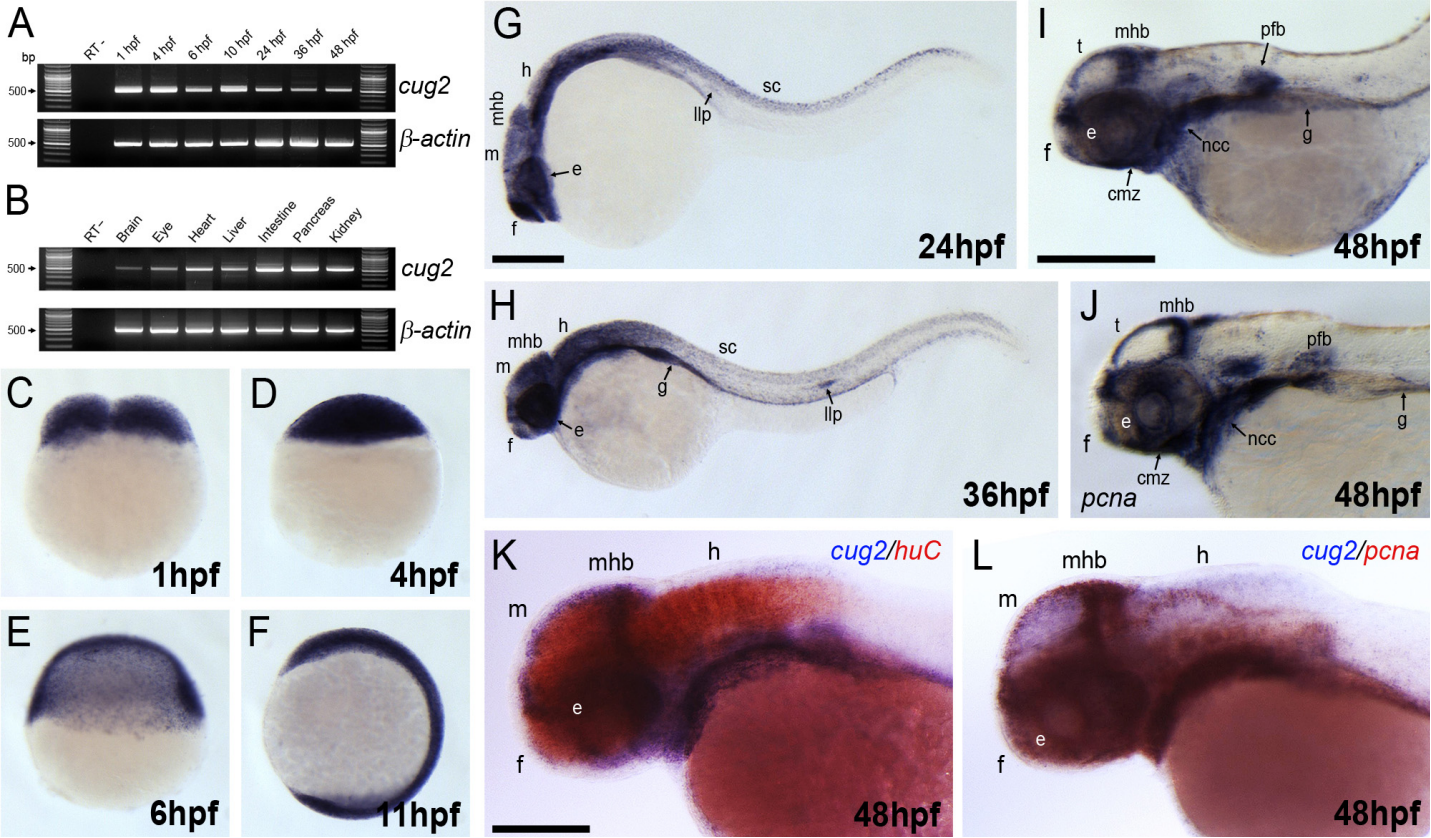
**Expression pattern of *cug2 *in developing zebrafish embryos**. **A**. Temporal expression profile of zebrafish *cug2 *by RT-PCR. Zebrafish *cug2 *transcripts have maternal and zygotic expression. β-actin is the loading control. **B**. The expression of *cug2 *is detected in the brain, eye, heart, liver, intestine, pancreas, and kidney in adult zebrafish. **C-F**. In cleavage (**C**), blastula (**D**), gastrula (**E**), and segmentation stages (**F**), *cug2 *transcripts are ubiquitously expressed throughout the embryonic body. **G**. At 24 hpf, *cug2 *transcripts are detected in the eye (e), forebrain (f), midbrain (m), midbrain-hindbrain boundary (mhb), hindbrain (h), spinal cord (sc), and lateral line primordium (llp). **H**. Expression of *cug2 *detected in the lateral line primordium, gut (g) and CNS at 36 hpf. **I**. Expression of *cug2 *in the ciliary marginal zone (cmz) in the eyes, tectum (t), midbrain-hindbrain boundary, neural crest cells (ncc), pectoral fin buds (pfb) and gut at 48 hpf. **J**. Expression pattern of *pcna *at 48 hpf. Scale bar = 200 μm. **K**. At 48 hpf, expression domain of *cug2 *(blue) is not overlapped with that of *huC *(red), a differentiating neuronal marker. Scale bar = 100 μm. **L**. *cug2-*expressing region is almost overlapped with *pcna*-expressing proliferating zones at 48 hpf.

To determine the temporal and spatial expression patterns of the *cug2 *gene during zebrafish development, stage- and tissue-specific RT-PCR and *in situ *hybridization were performed, revealing that zebrafish *cug2 *transcripts have both maternal and zygotic expression patterns (Figure 2A). *cug2 *mRNA was detectable at variable levels throughout the early embryonic stages (1-48 hpf; Figure 2A) and in selected tissues in the adult (Figure 2B). Whole-mount *in situ *hybridization showed that *cug2 *transcripts were ubiquitously expressed throughout the embryonic body from cleavage to the early somite stage (Figure 2C-F). At 24-36 hpf, *cug2 *expression was specifically detected in the lateral line primordium, gut, and CNS including the telencephalon, midbrain, midbrain-hindbrain boundary, hindbrain, and spinal cord (Figure 2G, H). At 48 hpf, the *cug2*-expressing region overlapped with a proliferating cell marker, *pcna*-expressing region, such as those in the ciliary marginal zone in the eyes, tectum, midbrain-hindbrain boundary, neural crest cells, pectoral fin buds, and gut (Figure 2I, J, L). However, the expression domains of *cug2 *were not overlapped with that of *huC*, a differentiating neuronal marker (Figure 2K). These restricted and yet overlapping expression patterns indicate that *cug2 *is expressed mainly in the proliferating cell population of the CNS during early embryonic development in zebrafish.

### Knock-down analysis of *cug2 *in zebrafish embryos

**Figure 3:**
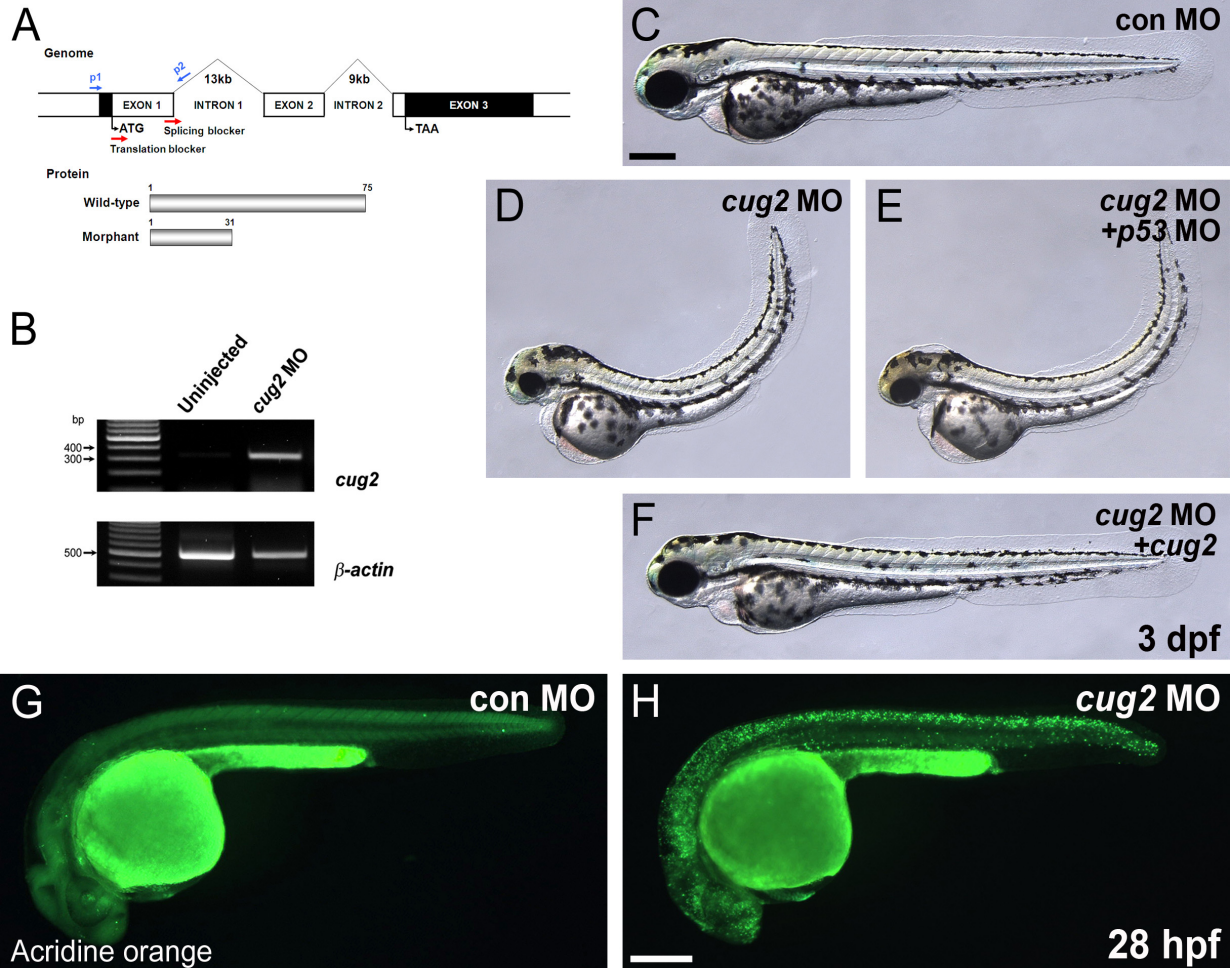
**Knock-down analysis of *cug2 *by morpholinos**. **A**. Genomic structure and MO targeting region (red arrow) of the zebrafish *cug2 *gene. **B**. Confirmation of the splice-blocking effect of MO by RT-PCR using p1-p2 primers (in A). Aberrant transcripts were amplified only in *cug2 *MO-injected embryos. β-actin is the loading control. **C-F**. In contrast to the control MO (**C**), *cug2 *MO-injected embryos (**D**) display neurodegenerative phenotypes including a flat head, small eyes, pinched midbrain-hindbrain boundary, thin yolk extension, and curved-up body at 3 dpf. Injection of *p53 *MO (**E**) did not affect these phenotypes in *cug2 *morphants, while co-injection of wild-type *cug2 *mRNA (**F**) rescued it at 3 dpf. **G, H**. Detection of cell death in CNS by acridine orange staining of *cug2 *MO- (**H**) and control MO-injected (**G**) embryos at 28 hpf. Scale bars = 250 μm.

To investigate the endogenous roles of *cug2*, knock-down analysis was performed using an antisense oligonucleotide morpholino (MO). The *cug2 *MO was designed to target the splicing donor site of exon 1, resulting in an aberrant transcript with a premature stop codon at residue 31 (Figure 3A). RT-PCR confirmed that injection of the *cug2 *MO into zebrafish embryos successfully blocked the splicing of *cug2 *transcripts (Figure 3A, B). Also, we designed other MO to target the translation start site (Figure 3A) and then confirmed that the MO specifically inhibits the translation of *cug2-GFP *containing its targeting region (Additional File 1: Figure. S1C, D). After 3 dpf, *cug2 *MO-injected embryos consistently displayed a range of characteristic phenotypes including flat head, small eyes, pinched midbrain-hindbrain boundary, thin yolk extension, and curved body (Figure 3C, D, Additional File 1: Figure. S1A, B, E, F). This phenotype was rescued by co-injection of wild-type *cug2 *mRNA, but not by injection of *p53 *MO (Figure 3E, F), confirming that the observed phenotype is specific to the effect of *cug2 *knockdown and not due to *p53*-dependent cell death, a typical off-target effect of MO [8].

To further determine whether the phenotypes observed in *cug2 *morphants were caused by apoptotic cell death, *cug2 *MO-injected embryos were analyzed by acridine orange staining. Acridine orange-positive cells were clearly and broadly detected throughout the bodies of *cug2 *morphants at 28 hpf, particularly in the CNS including the eye, brain, and spinal cord (Figure 3G, H). These data suggest that the phenotypes induced by morpholino knockdown of *cug2 *result from induction of apoptosis during early embryogenesis.

### *cug2 *deficiency causes neurodegeneration

**Figure 4:**
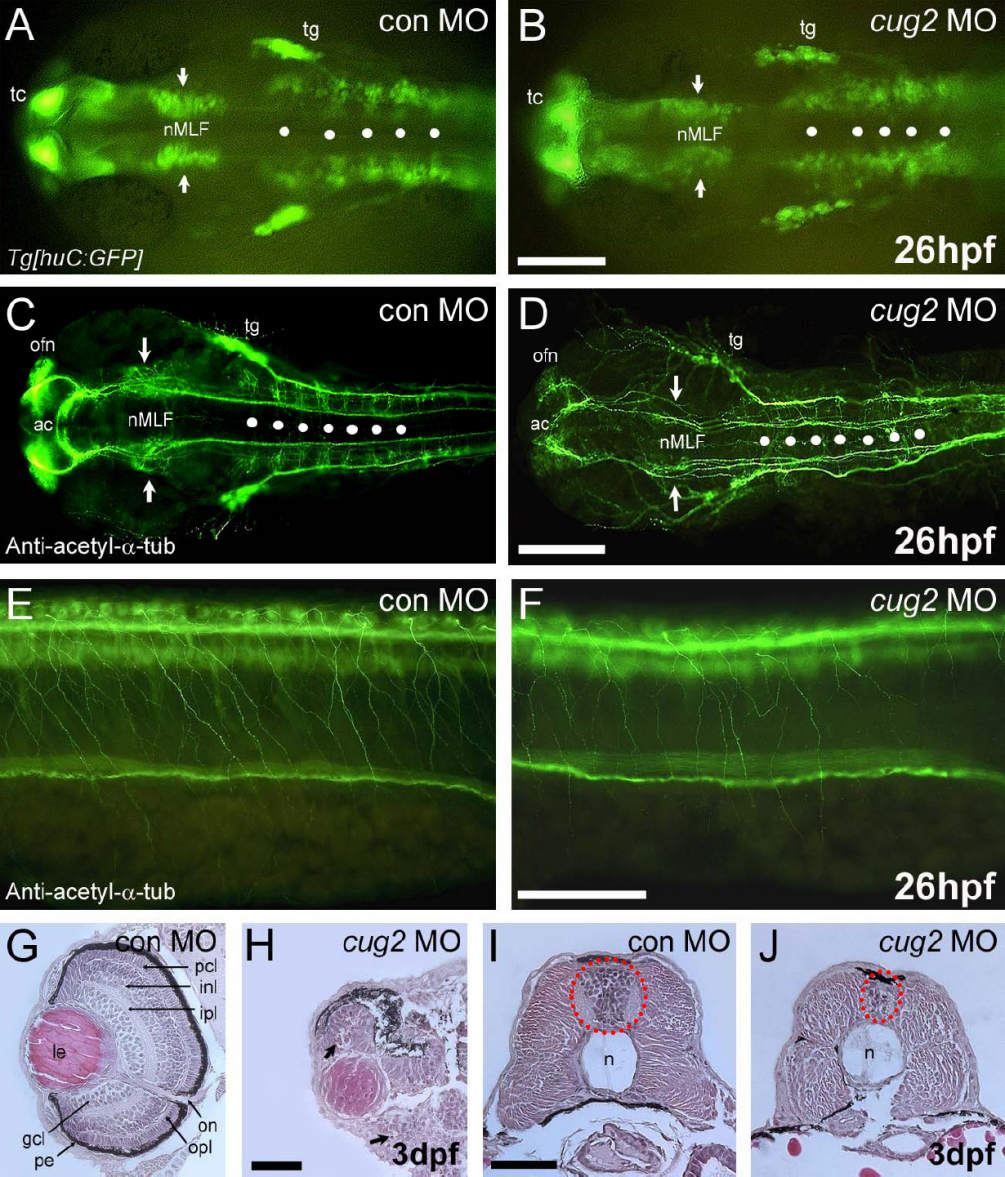
***cug2 *deficiency causes neurodegeneration in developing embryos**. **A, B**. In *huC:GFP *transgenic embryos, injection of *cug2 *MO (**B**) causes a reduction in the number of neurons in the nucleus of the medial longitudinal fasciculus (nMLF, arrows) and the rhombomere (white spots) compared to control MO (**A**). Scale bar = 200 μm. **C, D**. Anti-acetylated α-tubulin staining of the brain of control (**C**) and *cug2 *morphants (**D**). *cug2 *deficiency causes axonal scaffolding defects in the anterior commissure (ac), olfactory nerve (ofn), nMLF (arrows), and hindbrain commissure (white spots) at 26 hpf. Scale bar = 200 μm. **E, F**. Anti-acetylated α-tubulin staining of the spinal cord of *cug2 *morphants (**F**) at 26 hpf shows reduced arborization in Rohon-Beard (RB) sensory neurons compared to control (**E**). Scale bar = 100 μm. **G-J**. Histological sections of control (**G, I**) and *cug2 *MO (**H, J**)-injected embryos at 3 dpf. *cug2 *morphants exhibit severely disrupted retina layer formation (**H**) and a much smaller neural tube that contains fewer cells (**J**). gcl, ganglion cell layer; inl, inner nuclear layer; ipl, inner plexiform layer; le, lens; n, notochord; on, optic nerve; opl, outer plexiform layer; pcl, photoreceptor cell layer; pe, pigmented epithelium. Scale bars = 50 μm.

The phenotypes of *cug2 *MO-injected embryos are remarkably similar to previously reported neural degenerative mutants in zebrafish, such as *psm, terf2*, and *tub *[9-12]. Furthermore, the *cug2 *morphants exhibited extensive apoptotic cell death in the CNS (Figure 3G, H). Therefore, we utilized the *Tg[huC:GFP] *transgenic line that expresses neuron-specific GFP under the control of the *huC *promoter [13] to determine whether *cug2 *knock-down causes neuronal degeneration. Microinjection of *cug2 *MO into *Tg[huC:GFP] *transgenic embryos caused a reduction in the expression of GFP-positive neurons in the CNS, particularly in the nucleus of the medial longitudinal fasciculus (nMLF) and neurons of the hindbrain rhombomere at 26 hpf (Figure 4A, B). Moreover, the number of *huC*-positive differentiating neurons was dramatically decreased in *cug2 *morphants (Additional File 2: Figure. S2G, G'). Next, embryos were immunostained with anti-acetylated α-tubulin to investigate whether *cug2 *morphants display architectural defects of the axonal scaffold in the brain. As expected, the axonal scaffolds in the anterior commissure (ac), olfactory nerve (ofn), and nMLF were significantly deteriorated, as evidenced by significantly weaker acetylated α-tubulin staining compared to control. Particularly, organization of the commissural axons in the rhombomere segments was severely disturbed in *cug2 *MO-injected embryos (Figure 4C, D). In addition, reduced arborization was evident in neurons such as the spinal cord Rohon-Beard (RB) sensory neurons (Figure 4E, F).

To further define the neurodegenerative phenotype at the histological level, hematoxylin-eosin (H&E) staining was performed on serial paraffin sections of the brain, retina, and spinal cord at 3 dpf. Like other vertebrates, the zebrafish retina consists of six layers (ganglion cell, inner plexiform, inner nuclear, outer plexiform, outer nuclear, and pigment cell layers) and contains six types of neurons and one type of glial cell [14]. At 3 dpf, *cug2 *morphants showed severe disruption in the layer formation and pyknotic cells in the retina compared to control embryos (Figure 4G, H). Moreover, in the spinal cord, both the number of cells in the neural tube and the size of the neural tube were dramatically decreased in *cug2 *MO-injected embryos (Figure 4I, J). Interestingly, *cug2 *deficiency appeared to affect specific subpopulations of the developing neurons; during primary neurogenesis (3 ss) in the neural plate, *ngn1 *and *delta*-positive neuronal precursors were slightly increased by *cug2 *knockdown, while the *huC/elavl3*-positive differentiating neurons were decreased (Additional File 2: Figure. S2A-C', H). In contrast, during secondary neurogenesis (20 ss), both neuronal precursors and differentiating neurons were affected (Additional File 2: Figure. S2D-F'). These results indicate that *cug2 *may function in normal differentiation and/or maintenance of neurons rather than early neuronal precursor determination.

### *cug2 *morphants exhibit mitotic defects

**Figure 5:**
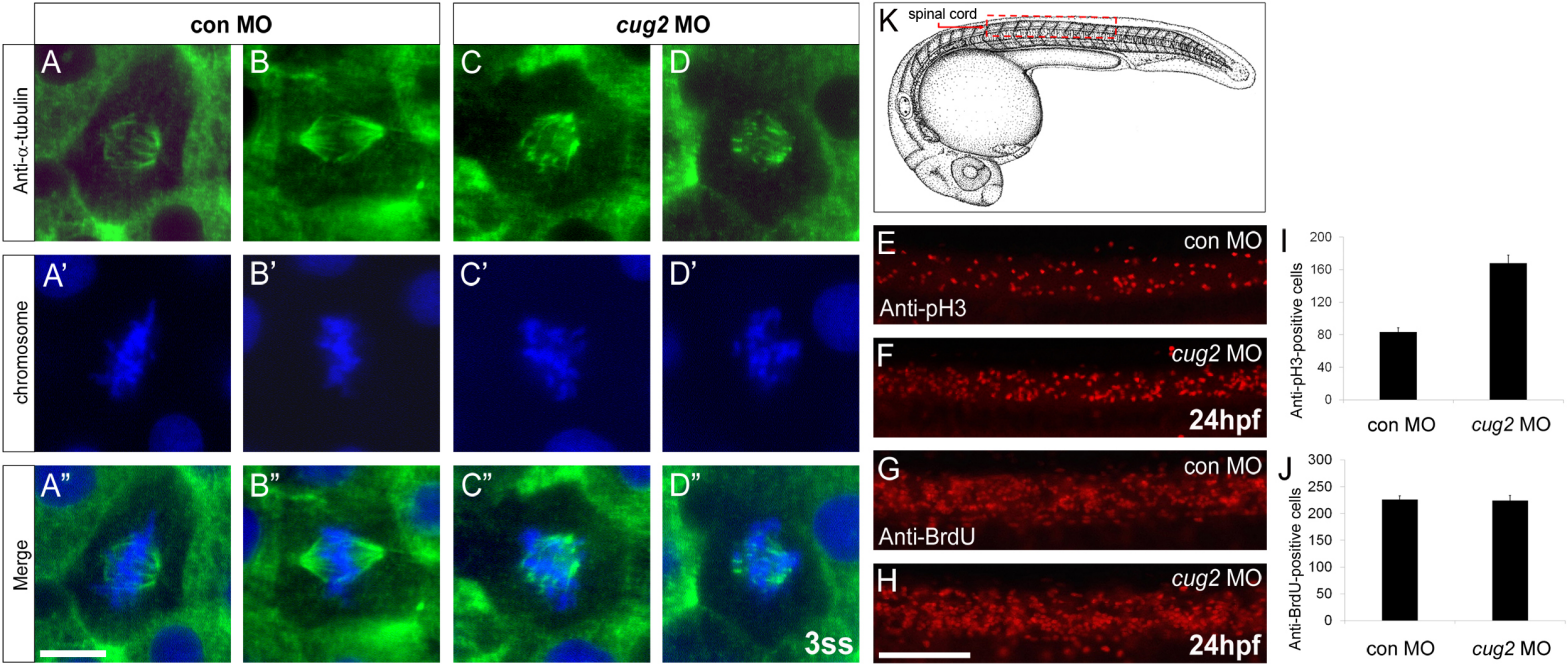
**Loss of *cug2 *function leads to defective mitosis**. **A-D"**. Anti-α-tubulin immunostaining of the control MO- (**A-A"**, **B-B"**) and *cug2 *MO-injected embryos (**C-C"**, **D-D"**) at the 3-somite stage. *cug2 *morphants display defective spindle formation and misaligned chromosomes at the metaphase plate (**C'**, **D'**). Scale bar = 10 μm. **E-H**. Lateral views of the spinal cord at 24 hpf. **E, F**. Anti-phospho-histone H3 immunostaining of control and *cug2 *morphants. **G, H**. Anti-BrdU immunostaining of control and *cug2 *morphants. Scale bar = 100 μm. **I, J**. Quantification of pH3- (**I**) and BrdU-positive cells (**J**) in control and *cug2 *morphants at 24 hpf (n = 10). The cells were counted from the trunk region, in an area of spinal cord schematically shown (red box) in **K**.

CUG2 was previously identified as a component of the constitutive centromere-associated network (CCAN) and abnormal mitotic events such as multipolar spindle formation and chromosome misalignment were observed in CUG2-depleted mammalian cells [2,3]. We first examined whether Cug2 localizes to cell's nucleus in zebrafish embryos, using GFP-tagged Cug2 protein. The behavior of Cug2-GFP is reminiscent of the behavior of the chromosomes during various stages of cell cycle in living zebrafish embryo (Additional File 3: Figure. S3A-C), thus suggesting that Cug2 is involved in mitosis in zebrafish. To investigate whether *cug2*-deficient embryos exhibit any defects in mitosis, embryos were double-stained with anti-α-tubulin antibody and Hoechst 33342 dye at the neural plate stage to visualize the mitotic spindles and metaphase chromosomes, respectively. In *cug2 *MO-injected embryos, α-tubulin-positive spindles were short and disorganized compared to the control embryos (Figure 5A-D''). In addition, *cug2*-deficient embryos exhibited varying degrees of disorganized metaphasic chromosome alignment (Figure 5'A-D').

Next, *cug2 *morphants were examined to determine whether there were any changes in the number of metaphase cells. Immunostaining of the embryos with an antibody against phosphorylated histone H3 (pH3), a mitotic marker [15], revealed that *cug2 *morphants experienced a significant increase in the number of pH3-positive cells in the spinal cord at 24 hpf (Figure 5E, F, I). The increased number of pH3-positive cells in the *cug2 *morphants likely represents an increase in mitotic arrest, rather than enhanced cell proliferation, since there was no significant difference in BrdU incorporation between the control and *cug2 *morphants (Figure 5G, H, J).

Combined, these results support the notion that loss of *cug2 *function causes defective mitosis, leading to mitotic arrest during early neurogenesis. We speculate that zebrafish *cug2 *is required for the normal function of the mitotic spindle and chromosome arrangement at the metaphase plate, consistent with the results from mammalian cell lines.

## Discussion

Here, we report that a zebrafish orthologue of a recently identified human centromeric protein CUG2/CENP-W is crucially important for normal mitosis and neurogenesis during early CNS development. Knockdown of *cug2 *expression in developing embryos caused a dramatic increase in the number of mitotically arrested cells exhibiting abnormal spindle formation and chromosome misalignment (Figure 5), as well as extensive apoptotic cell death associated with neurodegenerative phenotypes (Figures 3 &4).

We and others have previously shown that CUG2/CENP-W is a component of CCAN and participates in the formation of the DNA-binding complex together with CENP-A, CENP-C, and CENP-T at the kinetochore in mammalian cells [2,3,6]. Our current study further extends this notion and supports an essential role for zebrafish *cug2 *in kinetochore assembly, defects of which may elicit the checkpoint control mechanism and result in mitotic arrest. The genomic instability caused by the loss of *cug2 *affects cell viability, as evidenced by extensive apoptosis, leading to neurodegeneration early in CNS development in zebrafish. A number of studies in mice have shown that null mutations of the genes encoding most of the centromere proteins cause defective or arrested mitosis, and result in a degenerative phenotype and embryonic lethality [16-18]. In addition, genes encoding other zebrafish centromeric proteins, such as *cenpa/seph, cenpl *and *cenpn*, are mainly expressed in the proliferating regions during embryogenesis. Insertional mutation of these genes (*seph^hi2737Tg^, cenpl^hi3634Tg^*, and *cenpn^hi3505Tg^*) results in neurodegenerative phenotypes [19] similar to those of the *cug2 *morphants described in our study (Figure 3D).

Errors in chromosomal segregation due to compromised mitotic checkpoint control leads to aneuploidy, as often observed in transformed cell lines and human tumors. It has been postulated that common molecular pathways may be involved in both oncogenesis and neurodegeneration, and that genetic alterations of these pathways can lead to either carcinogenesis or neurodegeneration depending on the cellular context [20]. Considering the fundamental importance of genome stability in development, differentiation, growth and homeostasis of an organism, the data presented here support the critical role of CUG2 in both cancer and neurodegenerative diseases.

## Conclusions

In conclusion, this study suggests that Cug2 is required for normal mitosis during early neurogenesis and has functions in neuronal cell maintenance, thus demonstrating that the cug2 deficient embryos may provide a model system for human neurodegenerative disorders.

## Methods

### Fish stocks and maintenance

Adult fish were maintained at 28.5°C on a 14-hr light/10-hr dark cycle. *Tg[huC:GFP] *was used in knock-down experiments [13]. Embryonic stages were determined by the postfertilization hour and microscopic observation. Some of the embryos were treated with phenylthiocarbamide (1-phenyl-2-thiourea, PTU; Sigma) to suppress melanin synthesis. Animal work was approved by the internal animal ethics committee at Chungnam National University (No. 2010-3-6).

### Cloning of the zebrafish *cug2 *homologue

To isolate the zebrafish *cug2 *gene, a cDNA fragment from a 24 hpf zebrafish cDNA was amplified by RT-PCR based on the NCBI sequence (Genbank: XM_683789). The 519 bp PCR products were cloned into the pGEM-T easy vector (Promega, USA) and then subcloned into the *EcoR I *site in a pCS2+ expression vector. To construct the *cug2-GFP *fusion reporter into pCS2+ GFP expression vector, the specific enzyme-linked primers were designed for PCR amplification. PCR products were subcloned into the NcoI site in pCS2+GFP vector.

### Whole-mount in situ hybridization, immunostaining and apoptosis detection

To synthesis the RNA probe, a pGEM-T easy vector harboring the *cug2 *gene was linearized with *Sal I *and antisense *cug2 *RNA was transcribed *in vitro *using T7 RNA polymerase and digoxigenin or fluorescein-labeled UTP. The full-length cDNA of *pcna *(Genbank: AF140608) was amplified from a 24hpf cDNA and cloned into a pGEM T-easy vector. Antisense digoxigenin-labeled RNA probes for *ngn1 *[21], *huC/elavl3 *[22], *deltaA *[23], and *pcna *were produced using a digoxigenin-RNA labelling Kit (Roche, Germany) according to the manufacturer's instructions. Whole-mount *in situ *hybridization was performed as previously described [24]. Whole-mount immunostaining was carried out as described previously [24] using anti-α-tubulin, anti-acetylated α-tubulin, anti-BrdU (all from Sigma), and anti-phospho-histone H3 Ser10 (Cell Signaling) antibodies. For BrdU incorporation, dechorionated embryos were incubated with 10 mM BrdU in 15% DMSO/egg water (60 μg/ml sea salts (Sigma) in distilled water) for 20 minutes at 4°C and then for exactly 5 minutes at 28.5°C, followed by 4% paraformaldehyde fixation overnight at 4°C and dehydration in methanol at -20°C. To stain nuclei, the embryos were fixed in 4% paraformaldehyde, stained for 10 minutes with Hoechst 33342 (Sigma) and washed in PBS. For detection of apoptotic cells, embryos were placed in 10 μg/ml acridine orange (Sigma) diluted in egg water for 30 minutes and then washed in egg water.

### Paraffin sectioning and H&E staining

Embryos were fixed in 4% paraformaldehyde for 1 day at 4°C and then dehydrated with a graded ethanol series up to 100%. Specimens in xylene were embedded in paraffin and cut at a thickness of 6 μm. Histological hematoxylin-eosin (H&E) staining was carried out using standard protocols.

### Microinjection of mRNA and morpholino oligonucleotides

Synthetic capped mRNAs for human *CUG2*, zebrafish *cug2*, and *cug2-GFP *were transcribed *in vitro *by using the SP6 mMESSAGE mMACHINE Kit (Ambion). The synthesized mRNAs were dissolved in 0.2% Phenol Red as a tracking dye, and then microinjected into one to two cell stage embryos with 100 *pg *per embryo. Morpholinos were resuspended in 1 × Danieau's buffer (58 mM NaCl, 0.7 mM KCl, 0.4 mM MgSO_4_, 0.6 mM Ca(NO_3_)_2_, 5.0 mM HEPES, pH 7.6) with 0.1% phenol red and microinjected into embryos at the 1-4 cell stage. Concentration of morpholinos injected into embryos as follows: *cug2 *translation blocker, 500 *pg*/embryo; *cug2 *splicing blocker, control MO, and *p53 *MO, 2 *ng*/embryo. Injected embryos were incubated until the indicated stage and analyzed by *in situ *hybridization or immunostaining.

### DNA oligonucleotide and MO sequences

The RT-PCR primers used for cloning zebrafish *cug2 *cDNA from a 24 hpf zebrafish cDNA library were 5'-ataaaacgcctttcacgccgccaa-3' (forward) and 5'-gggctagatactgtccatcatcca-3' (reverse). The PCR primers for constructing *cug2-GFP *reporter were 5'- cgccatggggatgtcgtcagtaatctct-3' (forward) and 5'- cgccatggactgagtgtgtgtgtgtgca-3' (reverse). Morpholino antisense oligonucleotides for *cug2 *translation start site; 5'- CTG CTC TCG GTG CTT TCT TCG ACA T-3', the exon 1 splice donor site; 5'-GAA CCT TCT TCA ACT CAC CAT CAA G-3', standard control MO, 5'- CCT CTT ACC TCA GTT ACA ATT TAT A-3', *p53 *MO, 5'- TTG ATT TTG CCG ACC TCC TCT CCA C were designed to have no predicted internal hairpins, avoiding the presence of four consecutive G nucleotides, and synthesized by Gene-Tools, LLC (Corvallis, OR, USA).

## Authors' contributions

HTK, JHS, SHJ and WK carried out experiments. HTK, NSK, SL and CHK performed data analysis and participated in the design of the study. HTK, SHK, DGA, SL and CHK drafted the manuscript. SL and CHK conceived the study, participated in overall direction of the study. All authors read and approved the final manuscript.

## Supplementary Material

Additional file 1 **Phenotypes of *cug2 *translation blocking morpholino (ATG-MO) in zebrafish embryos**. **A, B**. *cug2 *MO-injected embryo (**B**) shows developmental defects including flat head, pinched midbrain-hindbrain boundary (arrowhead), thin yolk extension (arrow), and curved-up body. **C, D**. The translation blocking MO (ATG-MO) specifically inhibits the translation of *cug2-GFP *mRNA containing its targeting region. **E, F**. DIC image of PTU-treated *cug2 *morphant. The *cug2 *morphant shows retina degeneration and pinched brain structure (arrow) at 3 dpf. ov, otic vesicle; ret, retina. Scale bars = 200 μm. Click here for file

Additional file 2 **Early neurogenesis in *cug2 *morphant embryos**. **A, A'**. *neurogenin1 (ngn1*) expression in control MO- (**A**) and *cug2 *MO-injected (**A'**) embryos at the 3-somite stage (3 ss). The number of *ngn1*-positive neuronal precursors is increased at neural plate in *cug2 *MO-injected embryos (80%/n = 20). **B**, **B'**. Expression of *deltaA (dlA*) in control MO- (**B**) and *cug2 *MO-injected (**B'**) embryos at 3 ss. *cug2 *MO-injected embryos show increase of *delta A-*expressing neuronal precursor (70%/n = 20). **C, C'**. *huC *expression in control MO- (**C**) and *cug2 *MO-injected (**C'**) embryos at 3 ss. The number of *huC*-positive differentiating neurons is decreased in *cug2 *MO-injected embryos (77%/n = 26). **D-F'**. At the 20-somite stage (20 ss), *ngn1, deltaA*, and *huC *expression in control MO- (**D, E, F**) and *cug2 *MO-injected embryos (**D', E', F'**). During secondary neurogenesis (20 somite-stage), both neuronal precursors (*ngn1, delta A*) and differentiating neurons (*huC*) are decreased in *cug2 *MO-injected embryos. **G, G'**. Dorsal view at 24 hpf. The number of *huC*-positive differentiating neurons is dramatically decreased in *cug2 *morphants (**G'**). **H**. Quantification of *delta A, ngn1*, and *huC*-positive cells in control and *cug2 *MO-injected embryos at 3-somite stage in the area indicated in A-C' (n = 10). Click here for file

Additional file 3 **Subcellular localization of Cug2-GFP in zebrafish embryos**. Cug2-GFP protein (**A**) is co-localized with chromatin (**B**). Mitotic chromosomes are indicated by arrows (**C**). Scale bar = 30 μm. Click here for file
