# Supplementary figures and images for: Cug2 is essential for normal mitotic control and CNS development in zebrafish
Source: BMC Dev Biol. 2011 Aug 15;11:49. doi: 10.1186/1471-213X-11-49 (PMC3171718; doi:10.1186/1471-213X-11-49)

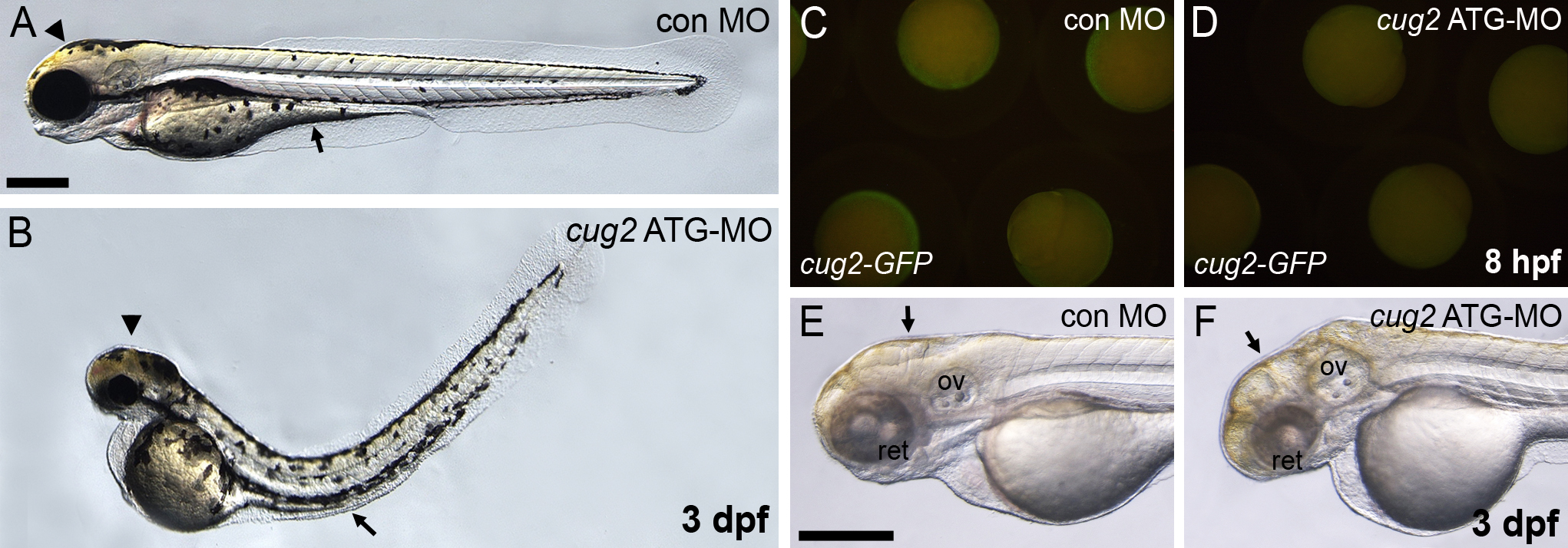

Supplement: Additional file 1 — Phenotypes of cug2 translation blocking morpholino (ATG-MO) in zebrafish embryos. A, B. cug2 MO-injected embryo (B) shows developmental defects including flat head, pinched midbrain-hindbrain boundary (arrowhead), thin yolk extension (arrow), and curved-up body. C, D. The translation blocking MO (ATG-MO) specifically inhibits the translation of cug2-GFP mRNA containing its targeting region. E, F. DIC image of PTU-treated cug2 morphant. The cug2 morphant shows retina degeneration and pinched brain structure (arrow) at 3 dpf. ov, otic vesicle; ret, retina. Scale bars = 200 μm. [file 1471-213X-11-49-S1.JPEG]

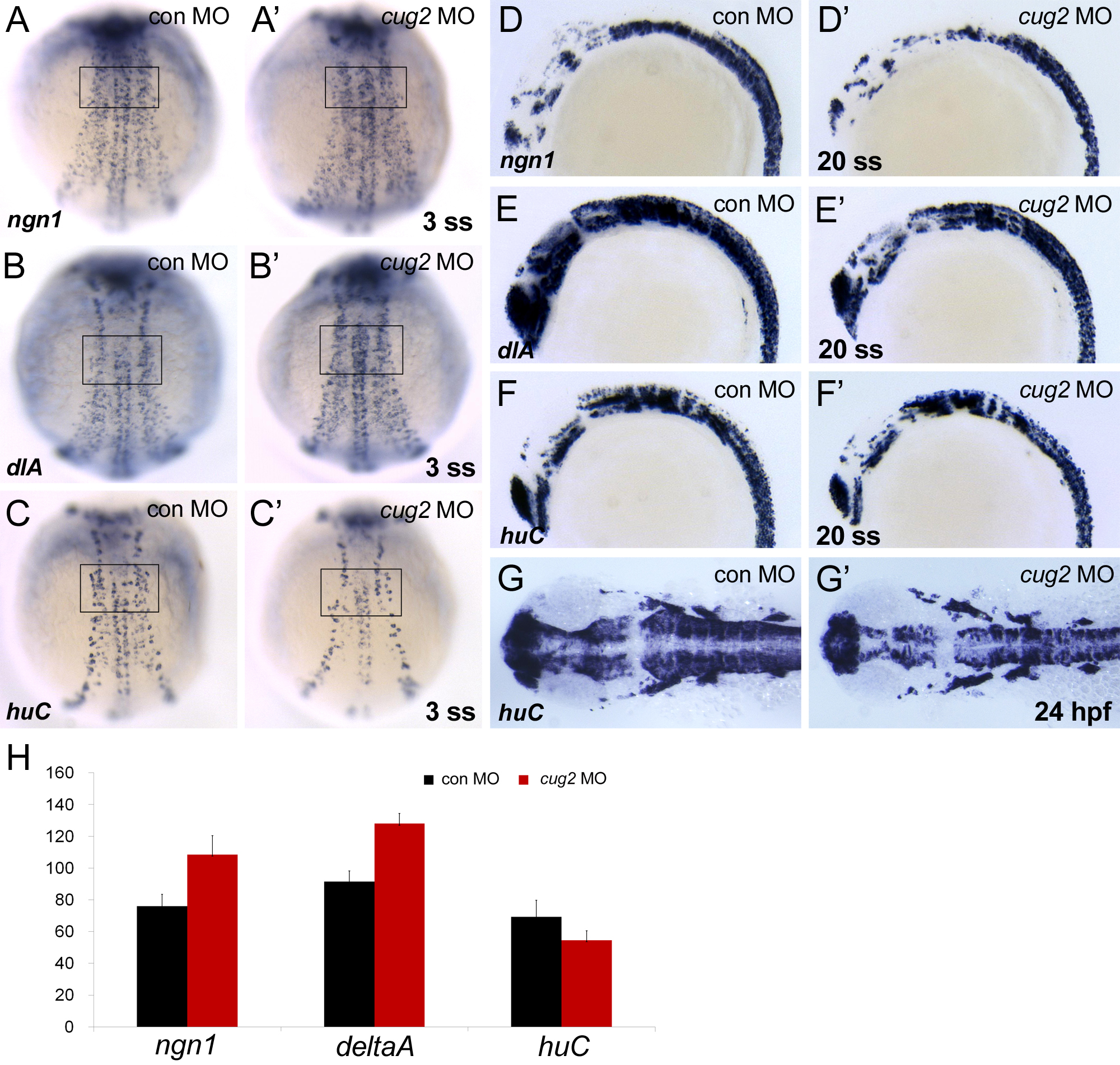

Supplement: Additional file 2 — Early neurogenesis in cug2 morphant embryos. A, A'. neurogenin1 (ngn1) expression in control MO- (A) and cug2 MO-injected (A') embryos at the 3-somite stage (3 ss). The number of ngn1-positive neuronal precursors is increased at neural plate in cug2 MO-injected embryos (80%/n = 20). B, B'. Expression of deltaA (dlA) in control MO- (B) and cug2 MO-injected (B') embryos at 3 ss. cug2 MO-injected embryos show increase of delta A-expressing neuronal precursor (70%/n = 20). C, C'. huC expression in control MO- (C) and cug2 MO-injected (C') embryos at 3 ss. The number of huC-positive differentiating neurons is decreased in cug2 MO-injected embryos (77%/n = 26). D-F'. At the 20-somite stage (20 ss), ngn1, deltaA, and huC expression in control MO- (D, E, F) and cug2 MO-injected embryos (D', E', F'). During secondary neurogenesis (20 somite-stage), both neuronal precursors (ngn1, delta A) and differentiating neurons (huC) are decreased in cug2 MO-injected embryos. G, G'. Dorsal view at 24 hpf. The number of huC-positive differentiating neurons is dramatically decreased in cug2 morphants (G'). H. Quantification of delta A, ngn1, and huC-positive cells in control and cug2 MO-injected embryos at 3-somite stage in the area indicated in A-C' (n = 10). [file 1471-213X-11-49-S2.JPEG]

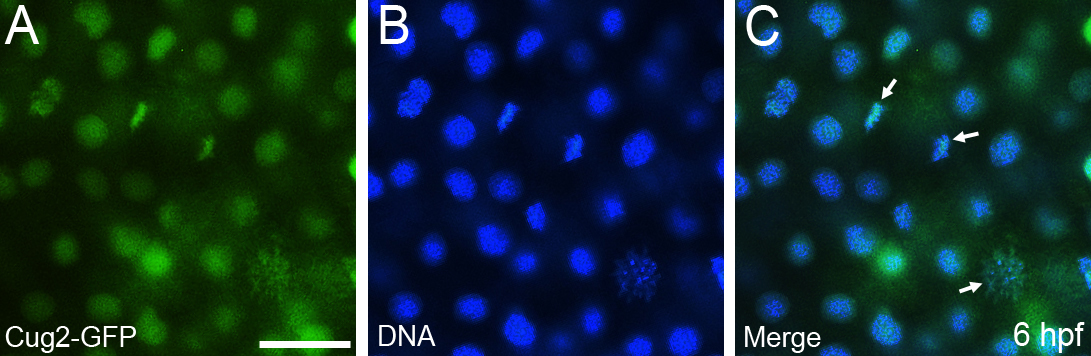

Supplement: Additional file 3 — Subcellular localization of Cug2-GFP in zebrafish embryos. Cug2-GFP protein (A) is co-localized with chromatin (B). Mitotic chromosomes are indicated by arrows (C). Scale bar = 30 μm. [file 1471-213X-11-49-S3.JPEG]
